# Using extreme risk protection orders to prevent violence among people experiencing homelessness in California and Colorado: a case series

**DOI:** 10.1186/s40621-026-00693-2

**Published:** 2026-07-02

**Authors:** Ari Davis, Rose Kim, Lisa B. Geller, Christopher E. Knoepke, Leslie Barnard, Garen J. Wintemute, Veronica A. Pear

**Affiliations:** 1 Center for Gun Violence Solutions, Johns Hopkins Bloomberg School of Public Health, Baltimore, MD USA; 2 Firearm Injury Prevention Initiative, University of Colorado, Denver, CO USA; 3 Centers for Violence Prevention, Department of Emergency Medicine, University of California, Davis, Sacramento CA USA

**Keywords:** ERPO, Violence, Homelessness, Qualitative, Firearm violence

## Abstract

**Background:**

Extreme Risk Protection Orders (ERPOs) temporarily restrict firearm access from those at risk of harming themselves and/or others. This case series describes when and how ERPOs have been used for people experiencing homelessness.

**Methods:**

We examined ERPO court case files previously abstracted in California (n = 37, 1/1/2016–6/30/2020) and Colorado (n = 12, 1/1/2020–12/31/2022) involving housing insecurity among respondents. We determined whether the respondent was experiencing homelessness (i.e., sleeping in a public place, shelter, vehicle, motel, or temporarily “couch surfing” with a friend). For each such case, we created narrative summaries and conducted thematic analysis.

**Results:**

We identified 25 ERPO respondents experiencing homelessness in California and 11 in Colorado; 83% (n = 30) were male, 44% (n = 16) were white, 25% (n = 9) were Black, and 22% (n = 8) were Hispanic. Domestic violence was mentioned in 44% (n = 16) of cases and a similar percentage (42%) mentioned suicide; 25% of cases involved threats of mass violence.

ERPOs were often filed in response to violence that occurred while respondents were losing their housing; reasons for impending homelessness included financial hardship, eviction, and loss of relationships. Other respondents experienced homelessness prior to the ERPO; threats of violence were intertwined with challenges of housing instability, loss of employment, family estrangement, untreated mental illness, and substance misuse. Serving ERPOs to people experiencing homelessness was challenging.

**Conclusions:**

ERPO respondents who were experiencing homelessness were in crisis and at high risk of violence. Courts and law enforcement agencies should consider how to improve serving ERPOs, recovering firearms, and providing services to homeless respondents.

**Supplementary Information:**

The online version contains supplementary material available at 10.1186/s40621-026-00693-2.

## Abstract (highlights)

- What is already known on this topic: Extreme Risk Protection Orders (ERPO) may prevent violence by temporarily restricting firearm access from those at risk of harming themselves and/or others. These orders are sometimes filed against people experiencing homelessness.
- What this study adds: We found that ERPO use for homeless individuals was rare in California and Colorado. Homelessness often contributed to the events leading to the orders’ issuance and posed challenges to ERPO implementation.
- How might this study affect research, practice, or policy: This study illustrates the challenges of using ERPO among people experiencing homelessness, including serving the order and recovering the firearms; it highlights the need for ERPOs to serve as a pathway to broader supports.

**Supplementary Information:**

The online version contains supplementary material available at 10.1186/s40621-026-00693-2.

## Background

People experiencing homelessness (PEH) face complex health and social challenges that increase the risk of suicide and interpersonal violence. These include economic hardship, exposure to violence, high levels of substance use, untreated mental illness, criminalization, discrimination, and involvement in the criminal justice system [1, 2]. As a result, PEH have suicide rates estimated to be ten times higher than the general population [3]. PEH are also at heightened risk of becoming involved in interpersonal violence, either as victims or perpetrators [4].

Extreme Risk Protection Order (ERPO) laws — also known as “Red Flag Laws” — may help reduce suicide and interpersonal violence among PEH by temporarily restricting firearm access when individuals are at elevated risk for violence. ERPOs are civil court orders that allow firearms to be temporarily removed from individuals who pose a significant risk of harm to themselves or others [5]. They have been found to prevent suicide among respondents and at the population level and may prevent interpersonal violence as well, though the evidence is mixed [6–11]. In all 22 states, the U.S. Virgin Islands, and the District of Columbia with an ERPO law, law enforcement can petition for an ERPO and, in many states, family or household members and other designated parties can petition as well. A judge reviews evidence of the respondent’s (the subject of the order) risk for violence and, if the burden of proof is met, may issue an order that temporarily prohibits firearm possession or purchase and permits law enforcement to remove firearms already in the respondent’s possession. Temporary orders typically last for a few weeks, after which time there is a hearing for the final order at which the respondent can appear. If the judge determines that there is ongoing risk, they will issue a final order, typically lasting 1 year (up to 5 years in California) [5].

ERPOs are sometimes filed against individuals facing a wide range of economic and social challenges, including homelessness. For instance, a study of California ERPO respondents from 2016 to 2018 found that 7 out of 202 ERPO cases involved respondents that were currently or formerly homeless or housing unstable [12]. In Washington state, various forms of housing instability contributed to the threats of violence that prompted an ERPO petition in the first years the law was in effect [13]. A Colorado study found that law enforcement’s ability to serve the order and remove firearms was impeded for one homeless respondent [14].

In this study, we seek to build on these insights by describing the use of ERPOs among people experiencing homelessness in California and Colorado – two states with strong gun laws and large populations of people experiencing homelessness [15, 16]. California’s ERPO law, called a Gun Violence Restraining Order, or GVRO, went into effect in 2016, and Colorado’s ERPO law took effect in 2020 [5]. Collectively, these states have issued thousands of ERPOs to address threats of suicides, interpersonal violence, and to prevent mass shootings [14, 17–19]. It is unknown how often ERPOs are being used for PEH or how housing status contributes to the crisis precipitating the order, impacts the ERPO process, or affects law enforcement’s ability to ensure that respondents do not have access to firearms. It is also not known whether biases against PEH and criminalization of homelessness contributes to differences in use of ERPO among PEH. This study uses ERPO court case files to explore the circumstances under which ERPOs have been used among PEH who are undergoing a crisis. By examining cases in multiple states, we aim to deepen understanding of the complexities involved in implementing ERPOs among this vulnerable population.

## Methods

### Study population and inclusion criteria

This study builds on prior research projects that involved collecting and coding ERPO court records in California (January 2016-June 2020) and Colorado (June 2020-December 2022). Teams from both states included a code to capture respondent housing insecurity. We reviewed cases coded as housing insecure to identify the subset of respondents who were homeless at the time the order was issued or were likely to be homeless shortly thereafter.

We used a broad definition of homelessness consistent with that of the federal Department of Education’s McKinney-Vento framework and with definitions adopted in Colorado and California [20–22]. Homelessness included individuals residing in public places, shelters, vehicles, motels, as well as those who were temporarily staying with others because of housing loss, instability, or economic hardship ( “couch surfing” or doubled up). Consistent with federal homeless definitions, we also included cases where the respondent’s homelessness was imminent (within 14 days). These cases included ERPOs filed during a pending eviction or in response to domestic violence incident, where a respondent would be prohibited or unable to stay at their former residence and lacked secure housing. Respondents who were incarcerated when the ERPO was served but were previously homeless, and likely to be homeless upon release, were also included, consistent with federal definitions of homelessness. The research team met routinely to discuss ambiguous cases and decide whether they met inclusion criteria.

This study was approved by the Institutional Review Board at Johns Hopkins Bloomberg School of Public Health. All data included in this study were available through public records requests made within each state; confidential information was redacted prior to being made available to the research team. The public was not involved in this study.

### Analysis

The research team reviewed and abstracted ERPO court documents and developed narratives for all cases meeting the inclusion criteria. We abstracted information pertaining to the characteristics of the respondent, the circumstances surrounding the ERPO, type of homelessness (initial, episodic, or chronic), and prior police contact. Initial homelessness was used when respondents were or were about to become homeless at the time the order was filed but did not appear to have a history of prior homelessness. Episodic homelessness was used when individuals appeared to fluctuate between periods of being homeless and having more stable housing, often with family members or in a treatment setting. Chronic homelessness was defined as cases where the individual had a history of being homeless and appeared to have been homeless for an extended period prior to the ERPO filing. Prior police contact was defined as any record in the ERPO case file of arrest, conviction, or being a suspect in criminal activity within the last 10 years. Only information included in the ERPO case file was included in this analysis. Descriptive data were stratified by state to identify potential differences.

Case narratives summarized the circumstances of the ERPO and contextual factors that may have contributed to the crisis. For each narrative, we aimed to include a description of the circumstances that led to the ERPO, including how the respondent’s housing status may have been related to the crisis.

The full seven members of the study team (AD, CK, LB, LG, RK, GW, VP) reviewed and abstracted information from court case files and met routinely throughout the study to discuss emerging patterns and themes. After reviewing initial narrative drafts, the research team developed a standardized set of domains to include in each narrative when the information was available. These domains included the role of perceived mental health challenges (including substance use) in assessing risk and issuing the ERPO, risk factors for violence including firearm access, and details about the ERPO implementation process, including challenges related to the respondent’s housing status.

We then revised and finalized case narratives using these domains and developed a matrix with domains as columns and the cases as rows to facilitate comparisons across cases. In addition, the study team identified themes from within each individual narrative in the supplementary materials. Through an iterative process of narrative review, matrix comparison and team discussion, we identified broader themes across cases.

We selected five illustrative cases that reflect themes identified within the broader set of cases, as well as and the diversity in the respondents’ housing situation, race, gender, and geography. All case narratives and the themes identified within each narrative are included in the supplementary materials.

## Results

We identified 36 cases involving respondents experiencing homelessness: 25 in California (of 967 abstracted) and 11 in Colorado (of 238 abstracted). Across both states, most ERPO respondents experiencing homelessness were male (83%) and over half (56%) were a race other than white (Table 1). Black respondents accounted for 28% (n = 7) of cases in California and 18% (n = 2) in Colorado. The average age of respondents experiencing homelessness was 42.5 years (SD: 10.8). Law enforcement filed nearly all petitions, with one household petitioner (4%) in California and two (17%) in Colorado. Cases were geographically concentrated in 2 urban centres with high uptake of ERPOs: 88% (n = 22) of California cases were filed in San Diego County and 82% (n = 9) of Colorado cases were filed in Denver.

**Table 1:** Characteristics of homeless ERPO respondents & cases

**Characteristic**	**California** **(n=25)**	**Colorado** **(n=11)**	**Total** **(n=36)**
Sex			
*Female*	3 (12%)	3 (27%)	6 (17%)
*Male*	22 (88%)	8 (73%)	30 (83%)
Race /Ethnicity			
*Asian*	2 (8%)	0 (0%)	2 (6%)
*Black*	7 (28%)	2 (18%)	9 (25%)
*Hispanic/Latino*	8 (32%)	0 (0%)	8 (22%)
*White*	8 (32%)	8 (73%)	16 (44%)
*Unknown*	--	1 (9%)	1 (3%)
Age			
*Mean (SD)*	41.3 (11.7)	45.6 (17.5)	42.5 (10.8)
Petitioner			
*Law Enforcement*	24 (96%)	10 (83%)	33 (92%)
*Non-Law Enforcement*	1 (4%)	2 (17%)	3 (8%)
Location			
*San Diego County*	22 (88%)		
*Other CA County*	3 (12%)		
*Denver*		9 (82%)	
*Other CO County*		2 (18%)	
Year Filed			
*2018*	2	--	2
*2019*	17	--	17
*2020*	6	3	9
*2021*	--	5	5
*2022*	--	3	3
***Total***	25	11	36

### Housing

ERPOs were filed in both California and Colorado against respondents facing various forms of homelessness (Table 2). Initial homelessness was documented in one-third of cases, involving respondents who were facing pending eviction or recent displacement from their residence at the time the ERPO was filed. Nineteen percent of respondents faced episodic homelessness, appearing to fluctuate between periods of being unsheltered or living in a car and times of more stable housing. The remaining 44% of cases involved individuals who appeared to have experienced chronic homelessness, living outside, in a vehicle, in a shelter or doubled up with friends for an extended period prior to the ERPO.

**Table 2:** Housing situation of ERPO respondents experiencing homelessness

	**California** **(n = 25)**	**Colorado** **(n = 11)**	**Total** **(n = 36)**
**Type of homelessness**			
* Initial (imminent or recent)*	8 (32%)	5 (45%)	13 (36%)
* Episodic*	6 (24%)	1 (9%)	7 (19%)
* Chronic*	11 (44%)	5 (45%)	16 (44%)
**Living location**			
* Kicked out or evicted (pending or recent)*	7 (28%)	3 (27%)	10 (28%)
* Doubled up (couch surfing)*	3 (12%)	0 (0%)	3 (8%)
* Shelter, transitional housing or hotel*	1 (4%)	4 (36%)	5 (14%)
* Vehicle (car, van, RV)*	6 (24%)	1 (9%)	7 (19%)
* Unsheltered (living on the streets)*	6 (24%)	1 (9%)	5 (14%)
* Unknown*	2 (8%)	2 (18%)	4 (11%)

### Case circumstances

Petitions noted mental health challenges in 58% of cases (Table 3); mental health concerns were more prevalent in Colorado (91%) than in California (44%). A mental health hold during the ERPO crisis was documented in 28% cases, and substance use (use of illicit drugs and/or alcohol) was mentioned in 47% of all cases—both more commonly in Colorado than in California. Prior police contact was documented in 67% of cases, including all Colorado cases and over half of California cases. Respondents had firearm access when the ERPO was issued in 67% of cases, with similar rates across both states.

**Table 3:** Case details of ERPO respondents experiencing homelessness

	**California** **(n = 25)**	**Colorado** **(n = 11)**	**Total** **(n = 36)**
**Context**			
* Mental Health Issue*	11 (44%)	10 (91%)	21 (58%)
* Mental Health Hold*	5 (20%)	5 (45%)	10 (28%)
* Substance Use*	10 (40%)	7 (64%)	17 (47%)
* Prior Police Contact*	13 (52%)	11 (100%)	24 (67%)
* Firearm Access*	16 (64%)	8 (73%)	24 (67%)
**Type of violence**			
* Domestic Violence*	14 (56%)	2 (18%)	16 (44%)
* Self-Directed*	8 (32%)	7 (64%)	15 (42%)
* Mass violence*	4 (16%)	5 (45%)	9 (25%)
* Other Interpersonal*	10 (40%)	2 (18%)	12 (33%)
**Outcomes**			
* Arrest*	10 (40%)	1 (9%)	11 (31%)
* Firearms Recovered*	7 (28%)	4 (36%)	11 (31%)
* Full Orders Issued*	11 (44%)	9 (82%)	20 (56%)

Threats prompting the ERPO included various types of violence that often co-occurred: domestic violence (44%), self-harm (42%), other forms of interpersonal violence (33%), and mass violence (25%). Thirty-nine percent of cases included multiple forms of violence, with the most frequent overlap involving self-harm and some form of interpersonal violence (33% of all cases). Cases of domestic violence overlapped with self-harm, interpersonal violence and mass violence in 13% of cases. California cases more frequently involved domestic violence and other types of interpersonal violence while Colorado cases more frequently involved threats of suicide and mass violence.

Law enforcement recovered firearms in at least 31% of cases, with a similar percentage in both states. Arrests occurred during the ERPO process in nearly one-third of cases (31%), more commonly in California than Colorado (40% vs 9%). Judges issued final orders in about half (56%) of cases, more often in Colorado (82%) than California (44%).

### Analysis of case narratives

Five illustrative case narratives (Table 4) highlight respondents’ complex intersecting challenges and represent the patterns observed across all 36 cases (included in the supplementary materials). They show how housing instability, a history of violence, mental health and substance use, and firearm access often intersect, leading to a crisis in which an ERPO was filed. They also demonstrate the implementation challenges that arise in serving ERPOs to PEH and in providing them adequate support. Information on whether respondents who were homeless or at imminent risk of homelessness received housing support or other services to address these underlying problems were not documented in the ERPO court case files.

**Table 4:** Select case narratives of ERPO respondents experiencing homelessness

Primary case characteristics	Summary
***Case 1: Eviction, perception of being wronged by system, threatened shootout with police***	In California, a 63-year-old Black man living in his deceased parents’ foreclosed home threatened a shootout with police attempting to serve eviction papers. Officers petitioned for an ERPO after the respondent sent a letter to the bank stating that he would shoot anyone who tried to evict him because he was the rightful heir to the property and his parents were victims of predatory lending. The temporary ERPO was granted, and six firearms were recovered (weapons presumed to be owned by his late parents). After the ERPO was served, the respondent was arrested for making criminal threats and given a mental health evaluation. He had a prior felony conviction for assault with a firearm in 2008. The respondent later contested the ERPO, arguing that he only spoke of making a citizen’s arrest on those trying to wrongfully evict him. These claims were supported by declarations from friends and neighbours. Due to the pending criminal case, the ERPO hearing was postponed seven times. The respondent did not attend the final hearing, and a final ERPO was granted. There is no documentation of the final order being served, but the respondent’s address on the order was listed as the same house from which he was presumably evicted.
***Case 2: Substance misuse, suicide and domestic violence threats during break-up***	In Colorado, a 35-year-old white man with a long history of alcohol misuse and suicidal ideation was staying at a hotel following a break-up and job loss. The precipitating event involved a domestic dispute during which the respondent threatened to kill his ex-girlfriend. When police arrived, the respondent acted erratically and threatened to shoot officers and provoke officers into killing him. He was taken into custody and later placed on a mental health hold. The respondent’s ex-girlfriend reported that the respondent had expressed a desire to “end it all,” with his mental and physical health deteriorating over several weeks. Police petitioned for an ERPO; a temporary order was granted, and two firearms were removed. However, the final ERPO was later dismissed after the respondent entered inpatient mental health treatment.
***Case 3: Chronic homelessness, mental health and substance use, challenges serving ERPO***	In California, the father of a 37-year-old white man petitioned for an ERPO after his son assaulted him. His son showed up unannounced at his parents’ home asking for his firearms that he believed were stored there. When the father refused, the respondent proceeded to choke his father and pushed him to the ground. The parents did not know where the respondent went and did not have a way to contact him. The respondent’s father reported that around a year prior, the respondent “quit his job, divorced his wife, abandoning his 2-year-old boy, sold his house and belongings and started drifting around”. The father stated that his son had been on drugs, moving between states, staying in the streets or at shelters where his veteran status could help him get a bed. He had been in and out of mental health hospitals and had stopped taking his prescribed antipsychotic medication. A temporary ERPO was issued in addition to an Emergency Protective Order for domestic violence, filed by law enforcement. The temporary ERPO was continued to permit law enforcement more time to find the respondent and serve the order. The ERPO was served to the respondent two months later at a hotel where he was staying. The final hearing was held, and a final order was issued. The respondent did not attend the hearing. Guns belonging to the respondent were stored at his ex-wife’s house (a handgun) and a friend’s house (an AR-15). There was no documentation that the guns were removed.
***Case 4: History of mental illness, previous suicide attempts and domestic violence***	In Colorado, law enforcement petitioned for an ERPO after a 35-year-old white woman was engaged in a domestic disturbance at the home of her estranged spouse. Although she no longer lived there, she entered the residence, stabbed holes in the walls, made suicidal statements, and removed firearms from her spouse’s safe before discarding them and fleeing. Police later found her in a nearby parking garage acting erratically. She told officers that she had attempted suicide by ingesting medication and was subsequently taken to a hospital and placed on a mental health hold. It was unclear where the respondent was residing at the time the ERPO was filed. Prior to the crisis, she had posted on social media that she did “not have a bed to sleep in” along with suicidal statements. The respondent had a history of multiple suicide attempts including surviving a self-inflicted gunshot injury two years prior. The respondent was also previously arrested for domestic violence and assault. She did not own firearms and there was no documentation of firearm recovery of the firearm reported in the incident. A final ERPO was issued.
***Case 5: Financial and personal crisis, chronic homelessness, threats of mass violence***	In California, law enforcement petitioned for an ERPO for a 47-year-old Hispanic man experiencing homelessness who had made threats of mass violence on social media and threats of suicide to his former employer. The respondent had a history of domestic violence and had recently violated a restraining order by approaching his ex-wife’s home, asking to see her and their daughters. In explaining why an ERPO was needed, law enforcement noted that the respondents’ mental condition increased the risk of a harmful encounter with law enforcement or community members. They also noted his “homelessness [was] a factor due to the fact that he may not be able to be located to be offered the correct assistance to help him deal with his personal crisis.” A temporary order was granted and served. At the court hearing, the respondent denied having access to firearms and made incoherent references to conspiracy theories; a final order was issued. There was no documentation of firearm recovery.

Five themes were identified across cases, often overlapping and intersecting with one another:

1. **Homelessness began at time of crisis**: Many individuals became homeless during a crisis, often following a breakup, domestic dispute, or eviction (Table 4, selected cases: 1, 2, 4). These moments of acute housing instability frequently coincided with threats of suicide, domestic violence, or threats of mass violence. In some cases, individuals facing eviction made threats of mass violence in response to feeling unjustly evicted or wronged by the system.
2. **Chronic homelessness contributed to crisis:** Other cases involved individuals who experienced chronic or episodic homelessness prior to the ERPO filing (selected cases: 3, 5). These included individuals who threatened violence toward ex-intimate partners, family members, and former employers. They also involved vague or unspecified violent threats, including threats of mass violence, coinciding with a broader mental health crisis. Crises occurred as respondents faced the compounding challenges of homelessness, persistent unemployment, family estrangement, untreated mental illness, and substance misuse.
3. **Mental health challenges and substance misuse were common**: Many respondents had a history of mental health challenges, including substance misuse, that appeared to contribute to the crisis which prompted the ERPO (selected cases: 1, 2, 3, 5). For some, untreated substance misuse intersected with relationship troubles and threats of violence against former partners. Other individuals appeared to be experiencing delusions, paranoia and psychosis, often acting erratically and making unspecified threats of violence.
4. **Domestic violence and prior police contact contributed**: Cases involved individuals threatening or inflicting violence against family members and intimate partners (selected cases: 2, 3, 4, 5). ERPOs were occasionally filed during an arrest for domestic violence, or in conjunction with a protection order. Some individuals had previously been incarcerated for domestic or other interpersonal violence.
5. **ERPOs commonly filed as safeguards**: ERPOs appeared often to be filed to ensure that respondents were swiftly separated from firearms, even if they were likely to become prohibited from firearm ownership through alternative mechanisms, such as involuntary commitment or a felony conviction. (selected cases: 1, 4, 5). Respondents were often involuntarily hospitalized at the same time the ERPO was filed, which can result in a firearm prohibition. Other respondents appeared to face concurrent criminal charges or have a criminal history that prohibited them from firearm access.

## Discussion

ERPOs have been used among people experiencing homelessness in California and Colorado in response to threats of self-directed and interpersonal violence, including mass violence and domestic violence. They were often filed during a crisis in which individuals were forced to leave their home, either through eviction, financial hardship, or a loss of a relationship. In other cases, respondents were homeless prior to the ERPO filing. Threats of violence were intertwined with housing instability, financial hardship, family estrangement, interaction with law enforcement, and mental health and substance misuse. These combinations of risk factors raise the level of perceived risk associated with cases, increasing the social and political acceptability of using ERPOs in an effort to prevent violence [23]. Findings indicate that ERPOs are being used in appropriate circumstances with PEH, however, such cases have unique implementation challenges and more can be done to connect homeless respondents to needed social services. Results can inform EPRO implementation with this vulnerable population across ERPO states.

Jurisdictions across California and Colorado have increasingly relied on policies that criminalize aspects of homelessness [24, 25]. This trend had been reinforced by a recent Supreme Court ruling allowing cities to punish people for sleeping in public places, growing political rhetoric linking homelessness to violence, and large-scale homeless encampments sweeps [26, 27]. Within this context, some might worry that ERPOs could be used to target and criminalize PEH, channelling them into the legal system rather than as a supportive tool to prevent violence [28]. Similarly, ERPOs might be applied to PEH in ways they would not be used among the general population, reflecting biases that associate homelessness itself with dangerousness, even when actual signs of risk for violence are minimal.

These worries were not substantiated here; we found that ERPOs appear to be used sparingly among PEH. The situations in which ERPOs were used among PEH largely resembled those of the general ERPO population in these states [17, 29]. They were used to prevent clear threats of firearm violence among a population that had exhibited risk factors for suicide and interpersonal violence.

In both states, PEH respondents resembled the larger ERPO population in prevalences of substance use and mental health challenges and in the petitioner type. In California, 40% of ERPO respondents experiencing homelessness and 34% of respondents generally reported substance use [17]. In Colorado, prevalences were 64% and 66%, respectively [30]. In California, 20% of ERPO respondents experiencing homelessness reported a mental health hold, levels comparable to the general ERPO population in California (23%) [17]. Mental health issues were more common among ERPO respondents experiencing homelessness in Colorado (91%) than the general population of respondents in that state (38%) [29]. However, the small sample size for PEH suggests this proportion may be unstable. Small sample sizes also make it challenging to determine if the types of petitioners among PEH respondents were related to case characteristics. However, our general findings appear to mirror petitioning trends, with law enforcement accounting for the vast majority of ERPO petitioners in California and a majority in Colorado [12, 29]. The demographic characteristics of homeless ERPO respondents differed from the general ERPO population, more closely resembling the homeless population overall. Black people comprise roughly 5% of the general population in both Colorado and in California, and roughly the same proportion of overall ERPO respondents [29, 31–33]. In our study, 18% (Colorado) and 28% (California) of ERPO respondents experiencing homelessness were Black, nearly matching the estimated racial composition of PEH within each state [34, 35]. Our findings likely reflect the racial inequities among those who experience homelessness, rather than evidence of racial bias in ERPO use.

Among PEH respondents, ERPOs appeared to be a necessary tool to mitigate harm but insufficient at providing support to address the underlying challenges they faced. The case narratives illustrated how the untreated and underlying challenges PEH faced were often, quite literally, out in the open, and the ERPO process appeared to do little to address these challenges. Our findings underscore the need for ERPOs to serve not only as a protective measure, but also as a bridge to care [36]. While this is particularly evident among PEH respondents, it also applies to the broader ERPO population, where mental health, substance misuse, and financial hardship may be less visible. These cases highlight the need for comprehensive interventions to stabilize and support individuals while the ERPO is in place, which should promote short and long-term safety.

ERPO cases among PEH reveal unique implementation challenges. The unstable nature of homelessness and lack of a fixed address made it difficult for law enforcement to locate and serve respondents, and to ensure that the places in which respondents were residing did not have firearms. This is particularly challenging in cases where individuals experience episodic homelessness or are “couch surfing” between multiple residences, shelters, or elsewhere. Hearing dates were often postponed and documentation regarding firearm recovery was often missing from case records. While prior research suggests that this is an issue for all ERPO respondents, it is of particular concern for respondents experiencing homelessness [17, 32, 33].

These case narratives also illustrate that some PEH often continue possess and access firearms despite lacking secure housing. The qualitative information documented in these cases helps fill an important gap in understanding firearm ownership, access, and storage practices among PEH. Prior research has documented high levels of firearm violence exposure and firearm access among certain homeless subgroups, including unhoused youth and veterans, yet little is known about firearm possession among the broader PEH population or how firearm access changes as PEH first fall into homelessness. Improved understanding of these dynamics could help public health and safety practitioners better support PEH who are in crisis and vulnerable to violence, including through interventions that temporarily limit firearm access, like ERPOs [37, 38].

Taken as a whole, these findings suggest that ERPO implementation among PEH must account for the instability and complexity of homelessness and be paired with violence prevention strategies that offer behaviour and social supports to PEH. Courts and law enforcement should consider the unique challenges PEH face when determining whether an ERPO is an appropriate tool. Implementers should create clear processes to serve ERPOs and other protective orders to PEH, ensuring that orders are enforced and the respondent is directed toward care. Respondents who are transferred to involuntary psychiatric holds should be referred to long-term wraparound care. More broadly, ERPOs should be integrated into a system of care so when an individual is served an ERPO they are simultaneously offered services designed to support them.

### Limitations

Information about these cases varied widely in public court records and often omitted details about how long the individual had been experiencing homelessness, where they were staying, and whether they had prior contact with public safety or social service providers. Follow-up information was rarely included, making it unclear whether social and housing services were provided, whether criminal charges were also filed, or other health or legal outcomes for respondents. Documentation of firearm surrender or removal was also often missing. This is not unique to respondents experiencing homelessness. Court documents reflect the perspective of law enforcement officers and petitioners. Their accounts are necessarily incomplete and may not include structural or personal stressors that contribute to the respondents’ behaviour and crises. Future research should include the perspective of ERPO respondents themselves. It should also investigate whether ERPO had their intended effect by examining whether respondents had subsequent instances of harm to self or others. Despite these limitations, this study provides rich information about the complex circumstances surrounding ERPO respondents experiencing homelessness.

## Conclusion

In California and Colorado, ERPOs among PEH appear to be filed in response to imminent threats of violence. Respondents made clear threats of harm to themselves or others and often had a documented history of behaving violently. Firearm access, or the perceived ability to access firearms, was listed in most of these cases, and often appeared—in conjunction with the threats of violence—to be the impetus for an ERPO. ERPOs were used in response to threats of suicides, mass shootings, and domestic violence; to protect parents, children, ex-partners and former employers, and the respondents themselves.

PEH are particularly vulnerable and could benefit from social services such as housing and employment assistance, mental health care, and substance use treatment. ERPOs can present an opportunity for respondents to be connected to such services, thereby addressing the underlying cause of the crises leading to the order, but it does not appear that ERPOs are being used in this way. Courts and law enforcement agencies should carefully consider how they can best serve ERPOs on, recover firearms from, and provide social services to ERPO respondents who are at acute risk for or are experiencing homelessness.

## Supplementary Information

Supplementary Material 1

## Data Availability

No datasets were generated or analysed during the current study.
