# Supplementary material for: Using extreme risk protection orders to prevent violence among people experiencing homelessness in California and Colorado: a case series
Source: Inj Epidemiol. 2026 Jul 2;13:51. doi: 10.1186/s40621-026-00693-2 (PMC13326436; doi:10.1186/s40621-026-00693-2)
Supplement: Supplementary file 1 — Supplementary Material 1 [file 40621_2026_693_MOESM1_ESM.docx]

**Supplementary Material for:**

**Using extreme risk protection orders to prevent violence among people experiencing homelessness in California and Colorado: a case series**

**Full List of ERPO Case Summaries**

***Selected Cases (Cases included in the manuscript)***

**Selected Case 1: California, Black male, age 63**

In California, a 63-year-old Black man living in his deceased parents’ foreclosed home threatened a shootout with police attempting to serve eviction papers. Officers petitioned for an ERPO after the respondent sent a letter to the bank stating that he would shoot anyone who tried to evict him because he was the rightful heir to the property and his parents were victims of predatory lending. The temporary ERPO was granted, and six firearms were recovered (weapons presumed to be owned by his late parents).

After the ERPO was served, the respondent was arrested for making criminal threats and given a mental health evaluation. He had a prior felony conviction for assault with a firearm in 2008. The respondent later contested the ERPO, arguing that he only spoke of making a citizen’s arrest on those trying to wrongfully evict him. These claims were supported by declarations from friends and neighbors.

Due to the pending criminal case, the ERPO hearing was postponed seven times. The respondent did not attend the final hearing, and a final ERPO was granted. There is no documentation of the final order being served, but the respondent’s address on the order was listed as the same house from which he was presumably evicted.

**Selected Case 2: Colorado, white male, age 35**

In Colorado, a 35-year-old white man with a long history of alcohol misuse and suicidal ideation was staying at a hotel following a break-up and job loss. The precipitating event involved a domestic dispute during which the respondent threatened to kill his ex-girlfriend. When police arrived, the respondent acted erratically and threatened to shoot officers and provoke officers to kill him. He was taken into custody and later placed on a mental health hold. The respondent’s ex-girlfriend reported that the respondent had expressed a desire to “end it all,” with his mental and physical health deteriorating over several weeks. Police petitioned for an ERPO; a temporary order was granted, and two firearms were removed. However, the final ERPO was later dismissed after the respondent entered inpatient mental health treatment.

**Selected Case 3: California, white male, age 37**

In California, the father of a 37-year-old white man petitioned for an ERPO after his son assaulted him. His son showed up unannounced at his parents’ home asking for his firearms that he believed were stored there. When the father refused, the respondent proceeded to choke his father and pushed him to the ground. The parents did not know where the respondent went and did not have a way to contact him.

The respondent’s father reported that around a year prior, the respondent “quit his job, divorced his wife, abandoning his 2-year-old boy, sold his house and belongings and started drifting around.” The father stated that his son had been on drugs, moving between states, staying in the streets or at shelters where his veteran status could help him get a bed. He had been in and out of mental health hospitals and had stopped taking his prescribed antipsychotic medication.

A temporary ERPO was issued in addition to an Emergency Protective Order for domestic violence, filed by law enforcement. The temporary ERPO was continued to permit law enforcement more time to find the respondent and serve the order. The ERPO was served to the respondent two months later at a hotel where he was staying. The final hearing was held, and a final order was issued. The respondent did not attend the hearing. Guns belonging to the respondent were stored at his ex-wife’s house (a handgun) and a friend’s house (an AR-15). There was no documentation that the guns were removed.

**Selected Case 4: Colorado, white female, age 35**

In Colorado, law enforcement petitioned for an ERPO after a 35-year-old white woman was engaged in a domestic disturbance at the home of her estranged spouse. Although she no longer lived there, she entered the residence, stabbed holes in the walls, made suicidal statements, and removed firearms from her spouse’s safe before discarding them and fleeing. Police later found her in a nearby parking garage acting erratically. She told officers that she had attempted suicide by ingesting medication and was subsequently taken to a hospital and placed on a mental health hold. It was unclear where the respondent was residing at the time the ERPO was filed. Prior to the crisis, she had posted on social media that she did “not have a bed to sleep in” along with suicidal statements. The respondent had a history of multiple suicide attempts including surviving a self-inflicted gunshot injury two years prior. The respondent was also previously arrested for domestic violence and assault. She did not own firearms and there was no documentation of firearm recovery of the firearm reported in the incident. A final ERPO was issued.

**Selected Case 5: California, Hispanic male, age 47**

In California, law enforcement petitioned for an ERPO for a 47-year-old Hispanic man experiencing homelessness who had made threats of mass violence on social media and threats of suicide to his former employer. The respondent had a history of domestic violence and had recently violated a restraining order by approaching his ex-wife’s home, asking to see her and their daughters. In explaining why an ERPO was needed, law enforcement noted that the respondents’ mental condition increased the risk of a harmful encounter with law enforcement or community members. They also noted his “homelessness [was] a factor due to the fact that he may not be able to be located to be offered the correct assistance to help him deal with his personal crisis.” A temporary order was granted and served. At the court hearing, the respondent denied having access to firearms and made incoherent references to conspiracy theories; a final order was issued. There was no documentation of firearm recovery.

***Additional Cases***

**Case 6: California, white male, age 31**

Law enforcement petitioned for an ERPO after a 31-year-old white man strangled his girlfriend and made suicidal threats during a breakup. The girlfriend reported that the respondent was living with her and that there had never been acts of physical violence before the incident. The respondent became physically violent after the girlfriend asked him “are you going to find a place to live?” The girlfriend reported that the only possessions the respondent owned in the house were clothes. She also mentioned that he had a history of mental illness. It did not appear that the respondent had a stable residence.

The girlfriend stated she would seek a restraining order but said she was hesitant to consider prosecution for assault. A temporary ERPO was granted, and the order was extended because the respondent could not be located to be served before the court hearing. One pistol was identified to be owned by the respondent. It does not appear that the respondent was ever served the temporary order, and neither party attended the final hearing. The case was dismissed.

**Case 7: California, Black male, age 32**

A 32-year-old Black veteran kidnapped his 6-year-old daughter and made violent threats towards the child’s mother and government officials. The ERPO was petitioned by law enforcement after respondent violated a custody order and kidnapped the child from school, attempting to take the child out of state. The respondent resisted arrest when police officers detained him. Police recovered one firearm during the arrest. An Emergency Protective Order for domestic violence was issued along with the temporary ERPO. The respondent did not attend the hearing where the final ERPO was granted.

The respondent was homeless and unemployed for an extended period and was sleeping in his car. He was separated from the child’s mother for several years and had limited contact with his daughter. Three months prior to the ERPO, the child’s mother secured a custody order citing his deteriorating mental health and an incident where he left town with their daughter for 10 days. The respondent himself, later spoke about his poor mental health stating to law enforcement that he was diagnosed with multiple mental health problems which impacted his health every day. He said he refused to seek care at the VA because it would be used against him in court.

**Case 8: California, Black male, age 33**

Law enforcement petitioned for an ERPO for a 33-year-old Black man who made violent threats and violated a protective order. The respondent had a history of multiple domestic violence incidents and was subject to current restraining orders. Law enforcement was contacted after the respondent told his ex-girlfriend, “I’m going back to jail and I’m going to kill myself, my one-year-old daughter [...] and my current girlfriend.”

The respondent was previously arrested at his girlfriend’s house and charged with violating a protection order and threatening a crime. The respondent reported that he was high on “spice” at the time of that incident. The respondent’s girlfriend reported that the respondent was homeless, mentally ill, used drugs, and had been violent.

Upon being informed of the threats, law enforcement responded to a support center for homeless adults where the respondent was known to frequent. The temporary ERPO was granted but the order did not appear to have ever been served. The petitioner requested a continuance of the temporary order twice to allow time for service. At the final hearing, the court dismissed the case. No firearms were recovered.

**Case 9: California, Asian male, age 55**

A 55-year-old Asian man was experiencing a schizophrenic episode and making threats while holding a BB gun in a parking lot. The respondent’s social worker arrived and told him to put the BB gun away because the police may mistake it as a real gun. The respondent replied, “they won’t take me alive” and “they should be worried, not me.” The respondent was transported by law enforcement to a hospital and put on a mental health hold. Six days after the incident, law enforcement petitioned for an ERPO.

The respondent stated that “I sometimes sleep on the street when I don’t live at my sister’s house.” He also mentioned that he has mental health problems and was not taking his medication. The respondent’s sister hadn’t seen the respondent for several weeks prior to the incident. No information was included in the court records about whether the respondent owned firearms.

A temporary order was granted and lasted for nearly a year due to multiple continuances related to the COVID-19 pandemic. The case was eventually dismissed because the respondent already had a firearms prohibition due to a criminal conviction. The respondent attended a continuance hearing but did not attend the final hearing. No firearms were recovered.

**Case 10, California, white female, age 51**

Law enforcement petitioned for an ERPO for 51-year-old white women who assisted her husband, a convicted felon, in threatening violence while armed. The husband, with the assistance of his wife, forced his brother into a tub of water to be “baptized” while putting a gun to his head. He and his wife then fled the home with multiple firearms including an AR-15.

A month prior to the incident, the respondent and her husband were living in Georgia when her husband believed that he needed to flee the state because CIA agents were coming to kill him. The couple drove to California to stay briefly with the respondent’s brother before being asked to leave. The incident took place shortly thereafter. Court documents state that the respondent was homeless.

A temporary ERPO was granted but was unable to be served because the respondent could not be located. Multiple continuances were granted while police monitored jail calls between the respondent and her in-custody husband and searched for the respondent’s car to issue her the order. The case was dismissed four months after the temporary order was initially granted because it was determined that the respondent moved out of state. No firearms were recovered.

**Case 11: California, Hispanic male, age 32**

A 32-year-old Hispanic man going through a divorce assaulted his wife, smashed her car, and stole her property. The man had alcohol and substance use problems and had recently lost his job. A month prior to the incident, the wife filed for a divorce, claiming that her husband was having an affair. She stated that her husband was “broke” and had been staying with friends. It was not clear where the respondent was staying after the incident, but it appeared he did not have stable housing or financial resources.

Police officers responded to the domestic violence incident and petitioned for a temporary ERPO and an Emergency Protective Order, both of which were granted. They encouraged the wife to file for a restraining order and she said she would do so, but there is no indication that she did. The temporary ERPO stated the man had access to multiple “unregistered and unidentified firearms.” No further details about the firearms were listed in the court documents. The temporary order was served, a final hearing held, and a final order issued. The respondent did not attend the hearing and there was no evidence that firearms were recovered.

**Case 12: California, white female, age 28**

A 28-year-old white woman threatened her on-and-off again girlfriend at gunpoint taking her girlfriend’s property and fleeing the apartment. The couple had a history of domestic violence in which the respondent’s girlfriend had previously been arrested. Ten days before the incident the respondent contacted her girlfriend saying that she was very sick and needed a place to stay temporarily. The respondent overstayed her welcome; she made her own key to the apartment and would frequently return to it in the middle of the night, unannounced and drunk. The incident occurred the morning after one of these episodes.

Law enforcement petitioned for an Emergency Protective Order for domestic violence and provided resources to the girlfriend. The girlfriend turned over the respondent’s shotgun, which was later determined to be stolen. In the months following the incident, law enforcement petitioned for and was granted a temporary ERPO and subsequently provided a continuance. The court later terminated the continuance stating that no reason for the continuance was given by petitioner. Hearings for the final order were postponed due to the COVID-19 pandemic. Six months after the incident, a hearing for the final ERPO was held in which the court dismissed the entire action per the petitioner’s request. There was no indication that the handgun used in the incident was recovered.

**Case 13: California, Black male age 36**

Law enforcement petitioned for an ERPO for a 36-year-old Black man after he assaulted his ex-girlfriend with a firearm. The respondent had recently been arrested but had no criminal history for domestic violence. He and his ex-girlfriend had broken up 10 days prior to the incident. They had been dating on and off for three months and had lived together in a motorhome before the respondent’s recent arrest. The incident began when the girlfriend returned to the motorhome and found the respondent taking property from it. Multiple individuals appeared to have access to the motorhome, including the respondent, who was notified that the motorhome was going to be towed the next day.

The respondent was arrested and charged for domestic violence battery. A temporary ERPO was granted, and two pistols were listed in the order. No firearms were located during the arrest or relinquished. The respondent did not attend the hearing, and a final order was issued. There was no documentation that the temporary or final orders were served to the respondent. The respondent address in the ERPO documents is listed as “unknown.”

**Case 14: California, Black male, age 42**

A 42-year-old Black man made threats of mass gun violence to medical staff after being denied information about the whereabouts of a patient. Threats were first made over the phone, and later, in person at the facility. The respondent was previously a patient at the facility and had become homeless since being discharged. Prior to making threats, he had been seen by staff sneaking onto the property to use the restroom and get cleaned up. The facility staff believed the threat to be credible and hired extra security as a result. One week later, the respondent was arrested after making threats at a different medical facility.

A temporary ERPO, petitioned for by law enforcement, was issued two months after the incident. The order stated that the suspect had access to guns, but there was no evidence that guns were retrieved. The hearing date was postponed due to COVID and multiple continuances of the temporary order were granted. The order was served to the suspect while he was at a psychiatric hospital four months after the temporary order was initially granted. The respondent did not attend the hearing for the final order, and full order was granted.

**Case 15: California, white male age 24**

A 24-year-old white man made threats of gun violence after he was told he had two weeks to move out of a family member’s house. The man was living in makeshift room set up in a garage. A family member called police after the respondent told her he would kill the whole family and that he had a gun. The responding officer reported that the respondent did not have a gun but that his father had multiple firearms locked in a gun safe, and his son did not know the code to the safe.

Law enforcement officers transported the respondent to a hospital for a mental health hold. Three weeks later, they petitioned for a temporary ERPO. A continuance of the order was granted because law enforcement was unable to serve the temporary order before the hearing date. The respondent was served the order at a restaurant where he worked. A hearing was held which noted that the respondent owned or was in possession of a firearm, and a final order was issued. The respondent did not attend the hearing and was served the final order afterword. There is no documentation of firearms being recovered.

**Case 16: California, Black male, age 53**

Law enforcement petitioned for a temporary ERPO after a 53-year-old Black man threatened to shoot a woman he had been stalking and harassing for several years. The victim had previously banned the respondent from her business for harassing the employees and customers. In the event which prompted the ERPO, the respondent entered into the victim’s business and stole items. After the victim ran after the respondent, he threatened her by saying “I’m going to get my gun and shoot you.” The victim believed the respondent was responsible for two previous burglaries at her residence. She had attempted to obtain restraining orders in the past, but the courts denied her requests. The victim believed the respondent was homeless and had previously seen the respondent with a handgun. A temporary ERPO was issued. Documents do not provide information about whether the respondent was served and if the hearing occurred, or firearms recovered.

**Case 17: California, Hispanic male, age 22**

Law enforcement petitioned for an ERPO for a 22-year-old Hispanic man with a history of perpetrating violence, after he sent threatening texts to a former co-worker after he was fired. Earlier that year, the respondent had battered a woman while she was waiting for the train. He was reportedly intoxicated during the incident and had been sleeping at the station for the past few weeks.

The respondent was known to stay at a residence during the weekends when he was not working; during the week he sometimes slept outside near his work. The respondent did not have registered firearms at the time the ERPO was filed but was reported to have been carrying a firearm in at least two separate instances reported to police. Prior to the ERPO, law enforcement recovered a firearm belonging to respondent at the property where he stayed on weekends; the firearm was returned to the respondent at the scene. A final ERPO was granted, and a notice was served to the respondent. Documents do not provide information about whether firearms were recovered.

**Case 18: California, Hispanic male, age 64**

Law enforcement petitioned for an Emergency Protective Order for workplace violence and a ERPO for a 64-year-old Hispanic man who made threats to shoot up his former place of employment. Prior to this, the respondent was placed on a mental health hold for making suicidal threats to his former employer’s director of HR. The respondent later demanded that his pension be increased and the medical bills for his mental health hold be paid by his former employer. He made a threat of mass violence which appeared to prompt the ERPO stating, “If I were to blow up or shoot up this place, I would have already. But I didn’t. I just want you to trust me.”

The respondent's housing status was unclear. On one ERPO form, the address listed was that of his sister’s, where he rented a room. Other notes describe him as “transient” and living out of a camper. Another police report noted that the respondent was depressed because he lost his kids and wife through divorce and was living on the streets. The temporary order was granted but there was no documentation of a final order or hearing, or of firearms being recovered.

**Case 19: California, white male, age 69**

Law enforcement petitioned for an ERPO after a 69-year-old white man made threats of suicide. A police officer was dispatched to the respondent’s address to investigate a report that a man had given his landlord a suicide note that stated, “this was not going to end well” and “this would end with him [the respondent] in a pool of blood.” The respondent was “stressed” because he “had not be able to pay rent.”

Law enforcement identified a handgun registered to the respondent, but he was not home when officers arrived on scene, and they were unable to locate him. When the respondent eventually called the officers back, he said he was meeting with his pastor when officers arrived and “was upset when he wrote that letter.” When asked about the firearm registered in his name the respondent stated that he possessed a firearm but that it was locked away in storage. As a result of COVID-19, multiple continuances to the temporary ERPO were granted. There is no documentation that a final hearing occurred, or that firearms were ever recovered.

**Case 20: California, Asian male, age 23**

Law enforcement petitioned for and were granted for an ERPO against a 23-year-old Filipino man who was found to be a threat to himself and others. Before the ERPO was filed, the respondent was placed on a mental health hold after telling an associate that he “would not be alive in the next five years” because “he was going to kill people and himself.” The respondent’s mother said he was currently homeless and living out of his vehicle. Police reports also showed that the respondent threatened to shoot his father and his ex-wife, but the respondent denied making these threats.

The petitioner requested multiple continuances for the temporary order stating that more time was needed to serve the notice because the respondent was homeless. A final hearing never occurred; no final order was issued and there is no documentation that firearms were recovered.

**Case 21: California, Hispanic male, age 38**

Law enforcement petitioned to renew an ERPO that was previously issued in 2019 and renewed in 2020. The initial ERPO was granted after a 38-year-old Hispanic man was arrested for intoxication and aggressive behavior towards his family members when they refused to hand him his loaded shotgun. When the first ERPO was issued, the respondent's firearm and ammunition were recovered. Although the ERPO was renewed for one year in 2020, the respondent continued to pose a significant danger to others; local police arrested him three additional times for public intoxication, vandalism, assault with a deadly (non-firearm) weapon, and witness intimidation. In addition, the respondent’s sister reported that the respondent was homeless and abusing drugs and alcohol. Further examination of the respondent’s criminal history revealed ongoing aggression and alcohol abuse that date back at least 16 years. The ERPO was renewed, and a 5-year order was issued.

**Case 22: California, Hispanic male, age 29**

Law enforcement petitioned for an ERPO after a 29-year-old Hispanic man threatened two people with a loaded firearm. Police responded to a call from an individual who witnessed the respondent pointing a firearm at a woman and a man. Police were able to locate the respondent who was living in his van. According to the respondent, he and a female friend agreed on a living arrangement in which the respondent would sleep on the friend’s couch for $100 every month. The living arrangement lasted for two weeks. The friend and respondent ran into each other, and a verbal dispute ensued because the respondent refused to pay after the friend burned his personal items. Law enforcement recovered an unregistered rifle and ammunition from the respondent’s van. A temporary ERPO was issued until the case was heard in court. The final ERPO was issued for one year.

**Case 23: California, white male, age 58**

Law enforcement petitioned for and were granted an ERPO after a 58-year-old white man made violent threats to harm his ex-wife. The respondent was brought to the hospital for suicidal ideation precipitated by personal events, including his divorce and house foreclosure rendering him homeless. In addition to his diagnoses of depression and anti-social disorder, the respondent also suffered from post-traumatic stress disorder and substance abuse. During his hospital visit, the respondent stated he was at the hospital because of his ex-wife, and that he wanted to hurt her. The respondent was released from the hospital to a homeless shelter. The hospital submitted a Tarasoff report based on a psychiatric-related incident, and a police officer and clinician were dispatched to investigate the incident. It was unknown if the respondent had access to weapons. A temporary ERPO was continued because the respondent could not be served. Documents do not provide information about whether the respondent was served, if the hearing occurred, or whether firearms were recovered.

**Case 24: California, white male, age 40**

Law enforcement petitioned to renew an ERPO that was previously issued in 2019, after a 40-year-old white man was arrested for the use of controlled substances. In 2019, a temporary and final ERPO were granted after the respondent yelled about shooting people at a public library. The respondent claimed he shot someone from his past and suffered from a post-traumatic stress episode due to his painful childhood. In addition, the respondent made multiple incoherent statements, and the respondent’s van also appeared to have incoherent scribblings written on the exterior. The police believed he suffered from a mental illness and posed a significant danger to himself and others. He was transported to the hospital where he was released to the care of the medical staff. The respondent lived out of his van. When the temporary ERPO and final ERPO were issued in 2019, the respondent's semi-automatic rifle and ammunition were recovered and remained impounded. The final ERPO was renewed for one year.

**Case 25: California, Hispanic male, age 46**

Law enforcement petitioned for an ERPO for a 46-year-old Hispanic man after he brandished a firearm during an argument with his ex-father-in-law. The ex-father-in-law was able to get the shotgun away from him and the respondent left the scene. The ex-father-in-law notified law enforcement and stated that he was fearful for his and family’s safety. Officers located the respondent’s abandoned van a few blocks away; inside were components of multiple firearms, which were recovered by law enforcement. This inciting event occurred 6 months before the ERPO was requested.

The respondent had multiple prior convictions related substance use and violence, including assault with a deadly weapon. He was listed as the suspect in 6 incidents of domestic violence against his ex-wife and identified to be a gang member. His address was listed as “transient; unknown address.” Officers believed he could be staying with his daughter or with fellow gang members, but they were unable to locate him. Documents suggest the case was being reviewed for criminal charges, but it is unclear if the respondent was ever charged or arrested. The temporary ERPO was granted. A continuance noted that the respondent had not been served. There was no indication that the final order hearing was held or that the final order was granted.

**Case 26: California, Black male, age 45**

Law enforcement petitioned for an ERPO for a 45-year-old Black man after he threatened his girlfriend with violence. The inciting event occurred about a month prior to the ERPO. The respondent left several threatening messages on his girlfriend’s voicemail after she refused to give him money from her tax return (e.g., “Lucky I didn’t put your teeth through your throat”). The respondent had been the suspect in a prior incident of domestic violence with the same woman and another incident with a different woman (8 years prior). The victim believed the respondent was a gang member and knew he carried an unregistered handgun in his backpack. Both the respondent and the victim were homeless. The police report was filed by the victim about a week after the event; the respondent was not arrested and criminal charges were not pursued. The temporary ERPO was granted, but two continuances noted that the respondent had not been served. There was no indication that a final ERPO hearing took place, that the final order was granted or firearms recovered.

**Case 27: California, Hispanic female, age 34**

Law enforcement petitioned for an ERPO for a 34-year-old Hispanic woman a month after she physically attacked her ex-boyfriend (the father of her child). The victim and respondent had ended their relationship prior to the attack and the respondent subsequently moved out of their shared home (but maintained ownership of it). Just prior to incident the respondent, heavily intoxicated, asked her ex-boyfriend to give her a ride to the house, but attempted to jump out of the vehicle on the freeway.

Once they arrived, the respondent punched her ex-boyfriend in the face and clawed at his neck and torso. Their 12-year-old daughter witnessed the altercation and called 911. She locked herself in the bathroom during the incident with her father’s pistol as a precaution, on the advice of the 911 operator. The respondent was arrested at the time of the event and charged with domestic abuse and child endangerment. She had a history of domestic violence with the same person. No firearms were registered to the respondent or the victim.

The temporary ERPO was granted but never served; as a result, no firearms were recovered, the final order hearing did not take place, and the final order was not issued. Three continuances for the final order hearing noted that officers were unable to serve the respondent with the temporary order but no additional detail was provided.

**Case 28: Colorado, white male, age 79**

Law enforcement petitioned for an ERPO for a 79-year-old white man living in an assisted living facility who had recently purchased a firearm and was threatening suicide. Police were requested by staff to do a welfare check on the respondent but determined he did not meet the criteria for an involuntary mental health hold. Later, the respondent’s firearm was found by staff and police were called again. He was evicted from the assisted living facility for violating the no-firearm policy and placed on a mental health hold; his firearm was recovered by police. The respondent attempted to live with a friend at another assisted living facility but was kicked out as that was against their policy. He relocated to a motel as temporary housing but was unable to pay for his room. He was also unable to go to any of his medical appointments due to lack of transportation, leaving him in chronic pain. As a result, the respondent ended up in the hospital, which provided him with resources to find housing and rehabilitation services where the police were contacted again and a temporary ERPO was issued and a final ERPO granted. At follow up, the respondent was living in another assisted living facility and was reportedly doing well, so the court-ordered psychological evaluation that was part of the ERPO was vacated, but the ERPO stayed in place.

**Case 29: Colorado, Black female, age 49**

Law enforcement petitioned for an ERPO for a 49-year-old Black woman after she made violent threats towards a crisis intervention program manager. The program manager reported that the respondent threatened her with a firearm and that she thought the respondent was dealing with untreated mental health issues exhibited by her erratic and paranoid behavior. The incident occurred while the respondent, her children and grandchildren were being evicted.

The respondent stated that there were many organizations, including a housing service provider and an internet provider, out to get her and she threatened a mass shooting in response. The crisis management team working to support the respondent said they had not previously seen her this emotionally unwell or exhausted. The respondent did not own firearms but stated a desire to access them. Police contacted the respondent and offered mental health services, which she refused. They subsequently petitioned for an ERPO; the temporary and final orders were granted.

**Case 30: Colorado, white male, age 38**

Law enforcement petitioned for and were granted a temporary ERPO for a 38-year-old white man after he threatened violence. The respondent was temporarily staying with his brother-in-law after being kicked out of his own home and facing financial troubles. The respondent’s brother-in-law called police to discuss a concerning display of behavior at his house; the respondent was not eating, and was abusing marijuana, methamphetamines, and cocaine. The brother-in-law described a conversation where he suggested the respondent seek mental health care and the respondent refused, saying he “hears voices and acts on what they are saying” and will be locked up because of it. Later the respondent threatened his brother-in-law via text message. The respondent had a history of child abuse, domestic violence, and investigations with Child Protective Services and was divorced from his spouse. The respondent repeatedly threatened his ex-wife despite a restraining order in place against him. The final ERPO was granted, and one firearm was recovered.

**Case 31: Colorado, white male, age 70**

The son of a 70-year-old white man petitioned for an ERPO for his father, who appeared to be suicidal. The son reported that the respondent was "talking to himself, not making sense" and threatening suicide or needing help because he “just wanted to die right now.” The respondent also stated that he wanted to hurt his wife and had recently purchased a pistol.

At the time of the ERPO, the respondent was residing in a hotel after having a fight with his spouse. The court documents include a long history of erratic communication within the family and describes the respondent’s ongoing medical challenges, including mental health and alcohol use, and relationship issues with his wife involving potential domestic violence. The temporary and final ERPOs were granted; there was no documentation of firearm recovery.

**Case 32: Colorado, Black male, age 29**

Law enforcement petitioned for an ERPO for a 29-year-old Black man who made mass shooting threats toward public transportation employees, security guards, and multiple businesses. Law enforcement was contacted after an incident in which the respondent was physically removed from a bus because he refused to wear a mask (mid-2020). The respondent believed that he was being discriminated against and threatened public transportation and security staff stating that he would “pull a knife” on them if something like this happened again. The respondent also stated that he would defend himself against “all police” who discriminate against him.

A few months after this incident, the respondent called multiple businesses threatening to come and shoot them. He also has a prior history with police after threatening to “shoot up” restaurants and making threats of suicide including “jumping in front of a train.” The respondent was documented to be homeless. While the temporary ERPO was granted, it was hard to serve documents to the respondent because he was homeless at the time. A full hearing was therefore delayed, but the final ERPO was eventually granted. There was no documentation of firearm recovery.

**Case 33: Colorado, white male, age 25**

A relative petitioned for and was granted temporary ERPO for a 25-year-old white man for threats of violence toward himself and others. The respondent had a history of bipolar disorder and often did not take medication as prescribed to treat his mental health issues. He had a history of manic/psychotic states, of resisting arrest, and at least 10 prior involuntary mental health holds. The petitioner indicated that the respondent was currently homeless.

Prior to filing the ERPO, the petitioner notified police of their concerns for reckless behavior and threats to self and others. The respondent was taken for an involuntarily mental health evaluation at the time the ERPO was filed. The respondent’s attorney was unable to contact the respondent and there were issues serving the temporary ERPO, which was eventually served. The court noted that respondent had no firearms in his possession, and the lawyer representing the respondent filed a motion to dismiss the final ERPO, which the court granted.

**Case 34: Colorado, male, unknown age and race**

Law enforcement petitioned for an ERPO for a homeless man of unknown age and race who made threats against police offers after an altercation. Police were called to a grocery store for reports of a man acting strangely and an altercation taking place. The respondent was brought to the hospital and placed on a mental health hold, where he tested positive for methamphetamine. He was known to police prior to this incident for other disturbances, drug use, and behavioral issues. At the precipitating event, the respondent threatened the police with a physical altercation and stated he wanted to "protect good people from bad police." Police searched the respondent's car (where he resided) and removed 2 rifles, approximately 1000 rounds of ammunition and a crossbow. The temporary ERPO was granted and then continued. Eventually, a final ERPO was granted.

**Case 35: Colorado, female, unknown race, age 61**

Law enforcement petitioned for and were granted a temporary ERPO for a 61-year-old women with undiagnosed and untreated mental health issues who was chronically (“for some time”) living on the street until being accepted into transitional housing. Staff of the transitional housing center called law enforcement after the respondent had discussed purchasing a firearm to shoot what she called "gas lighters" that no one can see but her. The manager was concerned about her mental health issues, repeated hallucinations, and disregard for the safety of other transitional housing residents. The respondent kept a knife under her pillow to protect herself from hallucinations. The respondent refused any mental health services offered by staff at the transitional housing complex. She had been previously arrested for threats of shootings and terrorism in the past, but she plead down her case so was still eligible to purchase a firearm legally. She did not appear to have firearm access at the time of the ERPO. A final ERPO was granted.

**Case 36: Colorado, White male age 35**

Law enforcement petitioned for and were granted a renewal of a final ERPO that was previously issued to a 35-year-old white man with a history of drug abuse, psychiatric holds, untreated mental health issues, suicidal threats, and mass shooting threats. The original ERPO stemmed from statements made to a former employer after the respondent was fired from his job. He threatened suicide with a firearm, saying he “wanted to end it,” and would die by suicide by cop if the police showed up. In seeking a renewal, law enforcement cited several incidents including the respondent’s verbal assaults to others, and threats of mass shootings at a retail store, both in person and over the phone. The respondent was reportedly using methamphetamine, alcohol, and other unknown substances. He appeared to be chronically homeless; in the original ERPO petition, the respondent was living at a campground, and seven firearms were recovered. The final ERPO was renewed.
